# COVID‐19 is associated with cardiac structural and functional remodelling in healthy middle‐aged and older individuals

**DOI:** 10.1111/cpf.12909

**Published:** 2024-10-08

**Authors:** Mushidur Rahman, Sophie L. Russell, Nduka C. Okwose, Gordon McGregor, Helen Maddock, Prithwish Banerjee, Djordje G. Jakovljevic

**Affiliations:** ^1^ Research Centre for Health and Life Sciences, Institute for Health and Wellbeing Coventry University Coventry UK; ^2^ Department of Cardiology University Hospitals Coventry and Warwickshire NHS Trust Coventry UK; ^3^ Research Centre for Healthcare and Community, Institute of Health and Wellbeing Coventry University Coventry UK

**Keywords:** cardiac structure and function, COVID‐19, speckle tracking echocardiography

## Abstract

**Background:**

Coronavirus disease 2019 (COVID‐19) was declared a global pandemic in 2019. It remains uncertain to what extent COVID‐19 effects the heart in heathy individuals. To evaluate the effect of the COVID‐19 on cardiac structure and function in middle‐aged and older individuals.

**Methods:**

A single‐centre prospective observational study enroled a total of 124 participants (84 with history of COVID‐19 [COVID‐19 group] and 40 without a history of COVID‐19 [non‐COVID group]). All participants underwent echocardiography with speckle tracking to assess cardiac structure and function at rest and during peak exercise.

**Results:**

There were no differences in left and right ventricular diastolic function (*p* ≥ 0.05) between the COVID‐19 and non‐COVID‐19 groups. Participants in COVID‐19 group demonstrated higher left ventricular mass (130 ± 39.8 vs. 113 ± 27.2 g, *p* = 0.008) and relative wall thickness (0.38 ± 0.07 vs. 0.36 ± 0.13, *p* = 0.049). Left ventricular global longitudinal strain was reduced in the COVID‐19 group at rest and at peak‐exercise (rest: 18.3 ± 2.01 vs. 19.3 ± 1.53%, *p* = 0.004; peak exercise: 19.1 ± 2.20 vs. 21.0 ± 1.58%, *p* ≤ 0.001). However, no difference was seen in resting left ventricular ejection fraction (58 ± 2.89 vs. 59 ± 2.51%, *p* = 0.565) between groups. Right ventricular fractional area change was reduced in the COVID‐19 group (*p* = 0.012).

**Conclusion:**

Cardiac structural and functional remodelling was observed in middle‐aged and older otherwise healthy individuals with a history of COVID‐19.

## 1 INTRODUCTION

Coronavirus disease 2019 (COVID‐19) is caused by severe acute respiratory syndrome‐coronavirus‐2 (SARS‐CoV‐2). The rapid‐spreading viral disease, predominantly targets the respiratory system in the form of pneumonitis (Li, Yang, et al., 2020), but is also known to affect the cardiovascular system (Zheng et al., 2020).

Myocardial injury is a common consequence of COVID‐19 which can lead to poor prognosis (Li, Guan, et al., 2020). COVID‐19‐infected patients with comorbidities such as hypertension, diabetes and underlying cardiovascular disease have an increased mortality risk up to threefold (Shi, Qin, Cai, et al., 2020; Wu & McGoogan, 2020). Histopathological evidence suggests viral particles are present in the myocardium causing low‐grade inflammation and acute coronary syndrome (Tavazzi et al., 2020). Myocarditis is caused by cytokine storm systemic hypoxia, coronary spasm, endothelial damage or through direct injury of the myocardium (Janus et al., 2020; Shi, Qin, Cai, et al., 2020). Ventricular failure may be a secondary effect of COVID‐19 with reduced left ventricular ejection fraction (LVEF) occurring in 8%–28% of patients with COVID‐19 (Chapman et al., 2020; Lippi & Plebani, 2020), potentially resulting in heart failure (Raisi‐Estabragh et al., 2022). The incidence of heart failure and concomitant death in patients with COVID‐19, is around 23% and 52%, respectively (Chen, Burdowski, et al., 2020; Zhou et al., 2020). Advanced, and more sensitive measurements of cardiac function such as speckle tracking echocardiography (STE) have been used to detect cardiac dysfunction in hospitalized patients (Shmueli et al., 2021).

The majority of previous research evaluating the effects of COVID‐19 on cardiovascular structure and function were conducted during acute COVID‐19 (Bhatia et al., 2021; Bleakley et al., 2020; Stöbe et al., 2020; Stockenhuber et al., 2020). The long‐term implications of COVID‐19 on cardiovascular structure and function remain unclear. The aim of the present study was to define the effects of COVID‐19 on cardiovascular structure and function in otherwise healthy middle‐aged and older individuals.

## 2 METHOD

### 2.1 Study design and patient population

**Figure 1:**
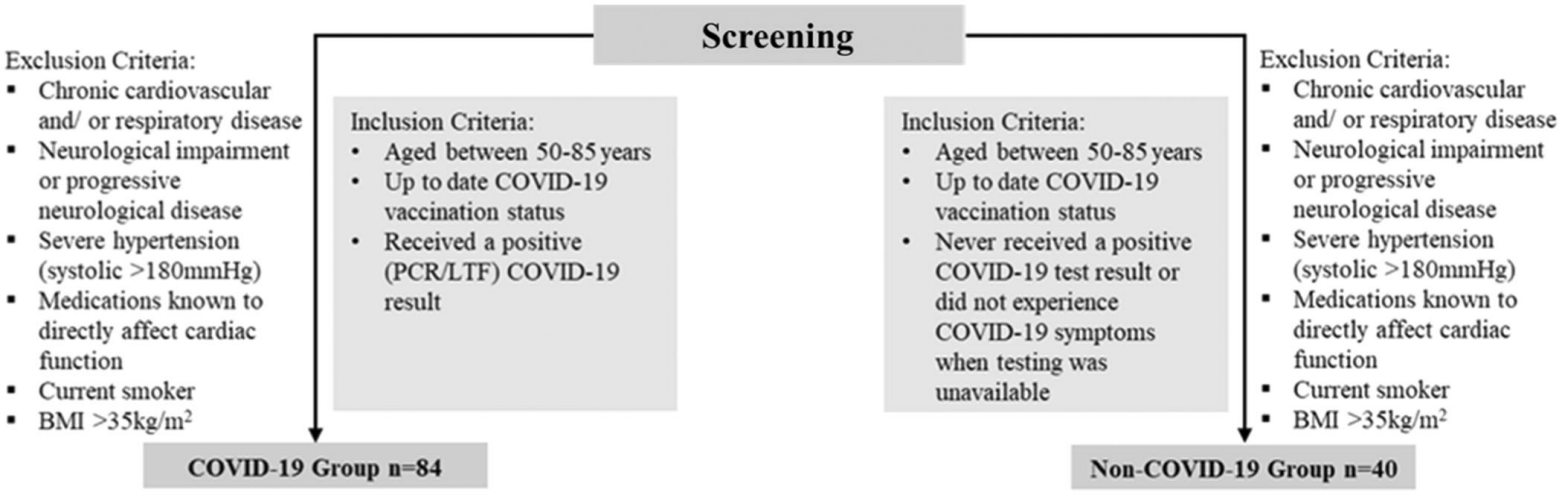
Flowchart of the study with inclusion and exclusion criteria.

This was a prospective, single‐centre, observational, study of 124 participants aged 50–85 years. Eighty‐four participants had a history of lateral flow test or polymerase chain reaction confirmed COVID‐19 (COVID‐19 group) and 40 participants had never tested positive or had COVID‐19 never had symptoms when testing was not available (non‐COVID‐19 group). The protocol was published in Trials (Rahman et al., 2023). Inclusion and exclusion criteria are outlined in Figure 1. None of the participants enroled were hospitalized with the infection however did suffer from mild COVID‐19 symptoms where fatigue (89%) and muscle aches (73%) were the most common. The study was performed in accordance with the Declaration of Helsinki and approved by Coventry University Research Ethics Committee (P125303) and the Health Research Authority National Health Service East Midlands—Leicester South Research Ethics Committee (22/EM/0090). Participants provided written informed consent.

### 2.2 Study assessments

Participants attended the Cardiovascular Research Laboratory at Coventry University to undergo assessments including anthropometry, blood pressure, 12‐lead electrocardiogram (ECG), echocardiography and maximal exercise testing.

### 2.3 Echocardiography

Echocardiographic examinations were performed using a commercially available machine (Vivid IQ, GE Healthcare) by an experienced, unblinded, accredited echocardiographer. Standardized transthoracic echocardiography was performed in accordance to the British Society of Echocardiography minimum data set (Robinson et al., 2020). All two‐dimensional and Doppler images were stored as cine‐loop images for a minimum of three consecutive beats. All measurements were made three times, and the average was stored. LEVF, end‐systolic and end‐diastolic volumes were calculated using the Simpson's biplane method (Gutiérrez‐Chico et al., 2005). Continuous Doppler was utilized to assess valvular function and calculate stroke volume (SV) and cardiac output (CO). Using pulse‐wave Doppler from the standard apical four‐chamber view, peak early (E wave) and late diastolic (A wave) filling velocities were used to calculate E:A ratio and mitral valve (MV) deceleration time for the LV and RV. Tissue Doppler images were used to calculate early diastole (e’) which was used to calculate mean E/e’. Right atrial (RA) pressure was evaluated based on size and collapsibility of the inferior vena cava. Dimensions for the left ventricle (LV), right ventricle (RV), aorta, left atrium (LA) and right atrium (RA) were measured using two‐dimensional (2D) images. In addition to standard measurements, LV stiffness index, LA stiffness index, RV diastolic function assessment and ventricular‐arterial coupling (VAC) assessments were made to assess LV systolic and RV diastolic function (Ikonomidis et al., 2019; Ngiam et al., 2020; Sugimoto et al., 2018; Zaidi et al., 2020). Methods and equations used for the calculation of echo parameters are contained in File S1.

Two‐dimensional greyscale images were analysed to make offline measurements for LV global longitudinal strain (LV‐GLS), LA strain (LAS) for contraction (LAct) and reservoir (LAres) phases and RV global longitudinal strain (RV‐GLS) using the EchoPac system (EchoPAC, GE Healthcare). A minimum of three cycles of high‐quality images at a frame rate between 40 and 90 frames/s were stored with optimal ECG quality. Apical four‐, three‐ and two‐chamber views were used to measure LV‐GLS. Apical two‐ and four‐chamber views were used to measure LAS. Apical four‐chamber view modified for optimal RV assessment was used to assess RV‐GLS (Dobson et al., 2021). Region of interest (ROI) was defined and automated tracking performed alongside visual assessment to ensure optimal myocardial tracking.

### 2.4 Stress echocardiography

LV‐GLS and LAS were measured during peak exercise on a semi‐recumbent cycle ergometer (Ergoselect 600). Doppler assessment in the apical five‐chamber view was utilized to calculate peak exercise SV and CO. Exercise testing was performed using a ramp protocol of either 10, 15 or 20 Watts/min depending on the individual. Patients were encouraged to maintain a cadence of approximately 60–70 revolutions per minute. Peak exercise was confirmed if heart rate reached ≥90% of maximal age‐predicted values. The Borg scale 6–20 was used for rating perceived exertion (Steeds et al., 2019).

### 2.5 Statistical analysis

Quantitative data were expressed as mean ± standard deviation (SD). Categorical data were presented as number (*n*) and frequency (%). A sample size of 124 participants would be required to detect a clinically meaningful relative difference of ~12.8% in LV‐GLS between the two groups with an intended power of 90%, a one‐sided α of 5% allowing an estimated attrition rate of ~10% (Lassen et al., 2020). Data were analysed for normality using the Kolmogorov–Smirnov test. Independent samples *t* tests were used for normally distributed data, and Mann–Whitney *U* tests for measurements without normal distribution. *χ* ^2^ tests were used for categorical data. Data were analysed using SPSS version 28 for Windows (SPSS Inc.). *p* < 0.05 was considered statistically significant.

## 3 RESULTS

A total of 124 (i.e., COVID‐19 group (*n* = 84; mean age: 60 ± 7 years; 55% females), and non‐COVID‐19 group (*n* = 40; mean age: 63 ± 7 years; 63% females) were enroled into the study. Baseline physical and clinical characteristics are provided in Table 1. Characteristics were similar for both groups except age (COVID‐19 group: 60 ± 7 vs. non‐COVID‐19 group: 63 ± 7 years, *p* = 0.023) and the presence of hypertension (COVID‐19 group: 18% vs. non‐COVID‐19 group: 23%, *p* ≤ 0.001). In the COVID‐19 group, the average time between the time of confirmed COVID‐19 and the date of visit was 221 ± 126 days.

**Table 1:** Baseline characteristics of the study population.

Participant characteristics	COVID‐19 group (*n* = 84)	Non‐COVID‐19 group (*n* = 40)	*p* Value
Participants (*n*) (%)	84 (68)	40 (32)	
Age (years)	60 ± 7	63 ± 7	0.023*^a^
Sex (females) (*n*) (%)	46 (55)	25 (63)	0.416^c^
Sex (males) (*n*) (%)	38 (45)	15 (38)	0.416^c^
Height (cm)	168 ± 9	166 ± 9	0.267^a^
Weight (kg)	76 ± 14	74 ± 14	0.331^a^
BMI (kg/m^2^)	26.9 ± 4.2	26.5 ± 4	0.591^a^
BSA (m^2^)	1.8 ± 0.21	1.8 ± 0.22	0.217^a^
Systolic BP (mmHg)	134 ± 17	130 ± 17	0.392^a^
Diastolic BP (mmHg)	83 ± 8	81 ± 10	0.156^a^
MAP (mmHg)	100 ± 10	97 ± 11	0.206^a^
Hypertension (*n*) (%)	15 (18)	9 (23)	<0.001*^c^
Diabetes mellitus (*n*) (%)	1 (1)	1 (3)	0.588^c^
Asthma (*n*) (%)	8 (10)	1 (3)	0.135^c^
*Ethnicity*			
White (*n*) (%)	76 (90)	39 (98)	0.159^c^
Asian or Asian British (*n*) (%)	7 (8)	1 (3)	0.216^c^
Black, Black British, Caribbean or African (*n*) (%)	0 (0)	0 (0)	
Mixed (*n*) (%)	1 (1)	0 (0)	0.488^c^

*Note*: * indicates statistically significant difference at *p* < 0.05.

Abbreviations: BMI, body mass index; BSA, body surface area; BP, blood pressure; MAP, mean arterial pressure.

^a^ Independent *t* test.

^b^ Mann–Whitney *U* test.

^c^ *χ* ^2^ test

### 3.1 Echocardiography

**Figure 2:**
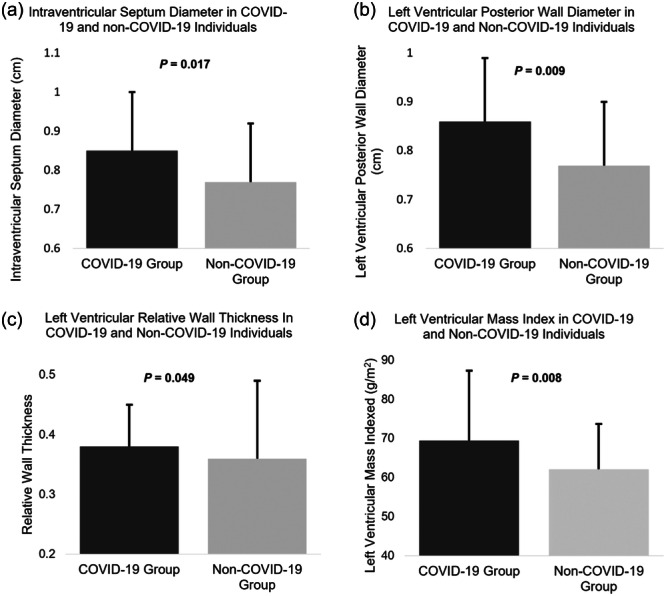
Significant differences in echocardiographic structural measurements: (a) Intraventricular septum diameter, (b) Left ventricular posterior wall diameter, (c) relative wall thickness, (d) left ventricular mass index between individuals in the coronavirus disease 2019 (COVID‐19) group and non‐COVID‐19 group.

Echocardiographic data are presented in Tables 2 and 3. At rest, LV‐GLS and LAS were not measured in six individuals from both COVID‐19 and non‐COVID‐19 groups, due to suboptimal image quality limiting tracking of the myocardial border. At peak exercise LV‐GLS and LAS were not measured in 20 participants and RV‐GLS and RV fractional area change (RVFAC) in one participant either due to diminished image quality or if the participant requested not to have stress echocardiography images taken. Due to there being a single observation, missing participant data for individual variables was removed from analysis and none of the data were included in any fitted models. There was a significant difference between COVID‐19 and non‐COVID‐19 groups in diastolic LV interventricular septum diameter (IVSd), posterior wall diameter (LVPWd), LV mass (LV mass) and relative wall thickness (RWT) (IVSd: 0.85 ± 0.15 vs. 0.77 ± 0.21 cm, *p* = 0.017; LVPWd: 0.86 ± 0.13 vs. 0.77 ± 0.20 cm, *p* = 0.009, LV mass: 130 ± 39.8 vs. 113.4 ± 27.2 g, *p* = 0.009 and RWT: 0.38 ± 0.07 vs. 0.36 ± 0.13, *p* = 0.049, respectively) (Table 2) (Figure 2). Participants in both groups had either normal LV geometry or concentric remodelling, however, there was no significant difference between the groups (*p* = 0.465).

**Table 2:** Comparison of structural echocardiographic measurements between COVID‐19 and non‐COVID‐19 groups at rest.

Structural echocardiographic measurements	COVID‐19 group (*n* = 84)	Non‐COVID‐19 group (*n* = 40)	*p* Value
IVSD (cm)	0.85 ± 0.15	0.77 ± 0.21	0.017*^b^
LVPWd (cm)	0.86 ± 0.13	0.77 ± 0.20	0.009*^b^
LVIDs (cm)	3.15 ± 0.58	3.15 ± 0.65	0.499^a^
LVIDd (cm)	4.56 ± 0.60	4.47 ± 0.64	0.678^b^
LV mass (g)	130.1 ± 39.8	113.4 ± 27.2	0.009*^a^
LV mass index (g/m^2^)	69.4 ± 17.9	62.1 ± 11.6	0.008*^b^
RWT	0.38 ± 0.07	0.36 ± 0.13	0.049*^b^
LV geometry			0.465^c^
Normal geometry (*n*)(%)	64 (76)	31	0.872^c^
Eccentric hypertrophy (*n*)(%)	0 (0)	0 (0)	
Concentric hypertrophy (*n*)(%)	0 (0)	0 (0)	
Concentric remodelling (*n*)(%)	20 (24)	9 (23)	0.872^c^
RV—base (cm)	3.49 ± 0.56	3.35 ± 0.44	0.319^b^
RV—mid (cm)	2.66 ± 0.49	2.48 ± 0.44	0.059^b^
RV—length (cm)	6.69 ± 0.37	6.68 ± 0.42	0.958^b^
LA volume (mL/m^2^)	23.4 ± 6.38	21.3 ± 5.44	0.112^b^
RA area (cm^2^)	7.94 ± 1.72	7.32 ± 1.56	0.058^b^

*Note*: * indicates statistically significant difference at *p* < 0.05.

Abbreviations: COVID‐19, coronavirus disease 2019; IVSD, intraventricular septum diameter in diastole; LA, left atrium; LVPWd, left ventricular posterior wall diameter in diastole; LVIDs, left ventricular internal diameter in systole; LVIDd, left ventricular internal diameter in diastole; LV, left ventricular; RA, right atrium; RWT, relative wall thickness, RV, right ventricle.

^a^ Independent *t* test.

^b^ Mann–Whitney *U* test.

^c^ *χ* ^2^ test.

**Table 3:** Comparison of functional echocardiographic measurements between COVID‐19 and non‐COVID‐19 groups at rest.

Functional echocardiographic measurements	COVID‐19 group (*n* = 84)	Non‐COVID‐19 group (*n* = 40)	*p* Value
Left ventricular ejection fraction (%)	58 ± 2.89	59 ± 2.51	0.565^b^
End‐systolic volume (mL)	45.3 ± 15.1	39.2 ± 9.71	0.048*^b^
End‐diastolic volume (mL)	108.1 ± 33.4	95.5 ± 23.4	0.075^b^
Global longitudinal strain (‐%)	18.3 ± 2.01	19.3 ± 1.53	0.004*^b^
Left ventricular stiffness index (mL^–1^)	0.001 ± 0.00	0.001 ± 0.001	0.089^b^
Stroke volume (mL)	76.6 ± 18.1	69.3 ± 17.1	0.021*^b^
Cardiac output (l/min)	4.6 ± 1.1	4.07 ± 0.84	0.015*^b^
Stroke volume index (mL/m^2^)	41.0 ± 8.00	38.0 ± 9.00	0.083^b^
Cardiac index (L/min/m^2^)	2.50 ± 0.50	2.30 ± 0.50	0.059^b^
Ventricular arterial coupling	0.60 ± 0.17	0.59 ± 0.18	0.494^b^
E/e’ average	7.80 ± 1.86	7.72 ± 1.49	0.724^b^
Diastolic function			
Normal diastolic function (*n*)(%)	49 (58)	23 (58)	0.900^c^
Grade I diastolic dysfunction (*n*)(%)	35 (42)	17 (43)	0.930^c^
Grade II diastolic dysfunction (*n*)(%)	0 (0)	0 (0)	
Grade III diastolic dysfunction (*n*)(%)	0 (0)	0 (0)	
RV‐FAC (%)	39.4 ± 5.94	42.2 ± 6.31	0.012*^b^
RV‐GLS (%)	21.1 ± 2.70	21.7 ± 3.21	0.482^b^
TAPSE (cm)	2.10 ± 0.31	2.03 ± 0.33	0.710^b^
RV S’ (m/s)	0.14 ± 0.02	0.13 ± 0.02	0.032*^b^
Diastolic function			
TV E:A ratio	1.22 ± 0.32	1.32 ± 0.24	0.142^b^
TV deceleration time (ms)	263 ± 56	262 ± 46	0.487^a^
Normal diastolic function (*n*)(%)	77 (92)	39 (98)	0.315^c^
Impaired RV relaxation (*n*)(%)	7 (8)	1 (3)	0.216^c^
Restrictive RV filling (*n*)(%)	0 (0)	0 (0)	
Reservoir (%)	29.1 ± 5.83	31.0 ± 6.21	0.100^b^
Contraction (%)	16.8 ± 3.63	17.4 ± 4.20	0.191^b^
LA stiffness index	0.22 ± 0.10	0.26 ± 0.07	<0.001*^a^

*Note*: * indicates statistically significant difference at *p* < 0.05.

Abbreviations: COVID‐19, coronavirus disease 2019; LA, left atrium; RA, right atrium; RVFAC, right ventricular fractional area change; RV‐GLS, right ventricular global longitudinal strain; RV S’, right ventricular systolic motion; TAPSE, tricuspid annular posterior systolic excursion; TV, tricuspid valve.

^a^ Independent *t* test.

^b^ Mann–Whitney *U* test.

^c^ *χ* ^2^ test.

**Figure 3:**
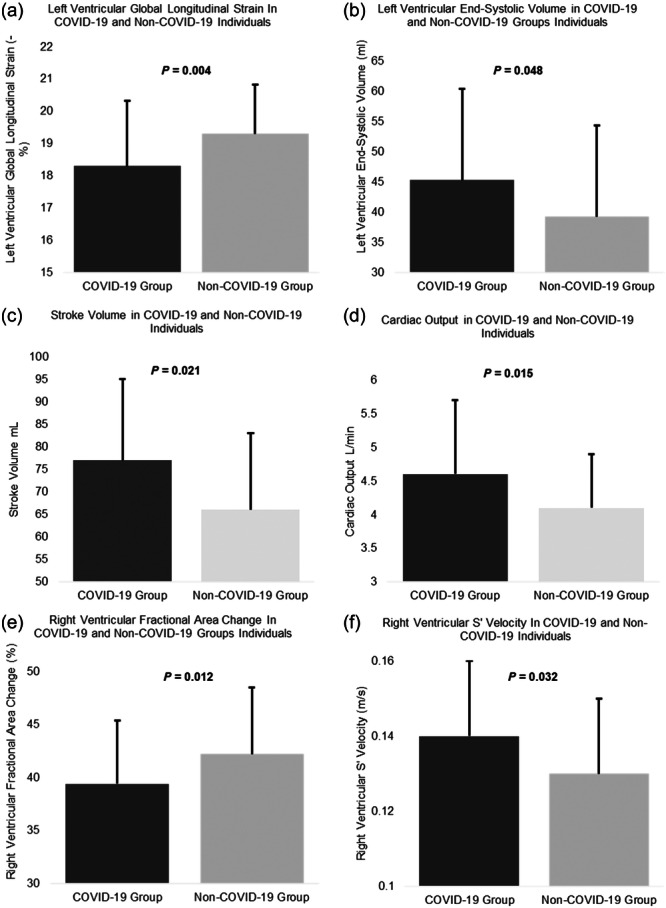
Significant differences in echocardiographic functional measurements (a) Left ventricular global longitudinal strain, (b) Left ventricular end‐systolic volume, (c) Stroke volume, (d) Cardiac output, (e) Right ventricular fractional area change, (f) Right ventricular S’ velocity between individuals in the coronavirus disease 2019 (COVID‐19) group and non‐COVID‐19 group.

Cardiac functional assessment revealed a significant difference between COVID‐19 and non‐COVID‐19 groups in measures of left ventricular function (end‐systolic volume: 45.3 ± 15.1 vs. 39.2 ± 9.71 mL, *p* = 0.048; SV: 76.6 ± 18.1 vs. 69.3 ± 17.1 mL, *p* = 0.021; CO: 4.60 ± 1.10 vs. 4.07 ± 0.84 L/min, *p* = 0.015 and LV‐GLS: 18.3 ± 2.01 vs. 19.3 ± 1.53 ‐%, *p* = 0.004) at rest. Those in the COVID‐19 group had significantly lower RVFAC compared with the non‐COVID‐19 group (39.4 ± 5.94 vs. 42.2 ± 6.31%, *p* = 0.012) and significantly higher RV s’ velocity than the non‐COVID‐19 group (0.14 ± 0.02 vs. 0.13 ± 0.02 m/s, *p* = 0.032), although these measurements are still within the reference ranges. Left atrial stiffness index was also significantly higher in the non‐COVID‐19 group than the COVID‐19 group (0.26 ± 0.07 vs. 0.22 ± 0.10, respectively, *p *≤ 0.001), these were within the normal reference range (Sugimoto et al., 2018). LV‐GLS was significantly lower in the COVID‐19 group compared with the non‐COVID‐19 group during peak exercise (19.1 ± 2.20 vs. 21.0 ± 1.58, *p* ≤ 0.001) (Table 4). No significant difference was demonstrated between the COVID‐19 and non‐COVID‐19 groups for right heart dimensions, LV and RV diastolic function (Table 2) (Figure 3). In addition, the proportion of participants with impaired.

**Table 4:** Comparison of function echocardiographic measurements between COVID‐19 and non‐COVID‐19 groups during peak exercise.

Echo measurements	COVID group (*n* = 84)	Non‐COVID group (*n* = 40)	*p* Value
Peak heart rate (bpm)	130 ± 19.3	122 ± 24.2	0.069
LV‐GLS (%)	19.1 ± 2.20	21.0 ± 1.58	<0.001^b^
LAS_Res_ (%)	37.5 ± 8.25	39.6 ± 5.76	0.215^b^
LAS_CT_ (%)	24.7 ± 6.56	24.7 ± 6.74	0.856^b^
Stroke volume (mL)	110 ± 31	106 ± 31	0.739^a^
Stroke volume Index (mL/m^2^)	59 ± 14	58 ± 18	0.896^a^
Cardiac output (L/min)	15.1 ± 4.5	16.3 ± 14.0	0.487^a^
Cardiac index (L/min/m^2^)	8.0 ± 2.0	8.8 ± 6.8	0.475^a^

Abbreviations: COVID‐19, coronavirus disease 2019; LAS_CT_, left atrial strain contraction phase; LAS_Res_, left atrial strain reservoir phase; LV‐GLS, left ventricular global longitudinal strain.

^a^ Independent *t* test.

^b^ Mann–Whitney *U* test.

## 4 DISCUSSION

The present study evaluated the cardiac structure and function in middle‐aged and older healthy individuals with and without a history of COVID‐19. We found that those with a history of COVID‐19 had significantly higher left ventricular wall thickness, LV mass and LA diameter compared to those without a history of COVID‐19. Those with a history of COVID‐19 also demonstrated significantly lower LV‐GLS and RVFAC at rest and LV‐GLS during peak exercise, however resting SV, CO and RV s’ velocity were significantly higher in the COVID‐19 group than the non‐COVID‐19 group. Despite lower mean LV‐GLS in the COVID‐19 group, a higher end‐systolic volume while maintaining a constant end‐diastolic volume could suggest the inadequate ejection of blood during systole (Støylen et al., 2020). However, the SV in the COVID‐19 group was also higher compared with the non‐COVID‐19 group which may suggest that due to reduced contractility (lower LV‐GLS), this maybe a compensatory mechanism to maintain overall cardiac function (Kerstens et al., 2024). This discrepancy may arise as a result of COVID‐19 which may not be uniformly affect all measures of cardiac function. This study provides valuable information that otherwise healthy individuals with a history of COVID‐19 may have subclinical myocardial damage. Since the emergence of COVID‐19, at least five other variants have been reported from the original wild‐type variant and have been reported as more transmissible (Vosko et al., 2023). Omicron variant was responsible for the most infections (Maslo et al., 2022). The impact of these variants on the cardiovascular system may differ. In the present study, the time between infection and assessments differed and this may potentially have influenced the findings.

Our study comprehensively assessed cardiac structure and function using conventional and advanced echocardiographic technology. Alongside conventional measures of cardiac function, our study also used novel parameters such as left ventricular stiffness index, left atrial stiffness index and left VAC to assess cardiac diastolic function and the interaction between the LV and the arterial system for efficient cardiac work. GLS is a sensitive tool that measures longitudinal shortening of the myocardium (Yang et al., 2018). The cut‐off values have been reported in previous literature and are technology dependent (Sugimoto et al., 2018). Observed differences in cardiac structural and functional measures between the two groups indicate there maybe physiological remodelling of the heart.

Since the emergence of the COVID‐19 pandemic, cardiovascular involvement has been demonstrated and has been associated with poor prognosis. Autopsy reports from an early study proved the presence of virus in cardiac tissue in 61.5% of subjects. A global echocardiographic evaluation of patients with COVID‐19 reported LV abnormalities in 39% of 479 patients, of which 3% had evidence of myocardial infarction and 3% of myocarditis (Dweck et al., 2020). Takotsubo cardiomyopathy was detected in 2% of the 479 patients (Dweck et al., 2020). Endocarditis and pericarditis were also detected in earlier studies (Dweck et al., 2020). This highlights the importance of cardiovascular screening and assessment of cardiovascular structure and function in those with a history of COVID‐19. Previous studies have shown that cardiovascular damage caused by COVID‐19 was predominantly seen in those with severe COVID‐19 symptoms which required hospitalization and mechanical ventilation. Myocardial injury was detected in up to 20%–30% of COVID‐19‐related hospitalization and 22%–32% of those being treated in intensive care unit (Oikonomou et al., 2022; Shi, Qin, Shen, et al., 2020).

Right heart structure and functional abnormalities were detected in 33% of patients in a previous study (Dweck et al., 2020). The present study demonstrates that those with a history of COVID‐19 have significantly reduced RVFAC compared to those without. Similarly, a previous study found that those with COVID‐19 had reduced RVFAC compared with healthy controls (Zhang et al., 2021). Chen et al. found RVFAC to be reduced in individuals over the age of 60 years (Chen, Wu, et al., 2020). Earlier studies reported RV dilatation in hospitalized patients with COVID‐19 and at follow‐up, 1‐month postdischarge which suggests RV dimensional abnormality to be persistent (Günay et al., 2021). However, RV dilatation was not apparent in the present study cohort and no significant difference was seen compared to the non‐COVID‐19 group. This could be because the present cohort was otherwise healthy whereas most participants hospitalized with COVID‐19 had pre‐existing comorbidities (Dweck et al., 2020). It could be also suggested that as this study was not conducted during the acute phase of the infection, acute dilatation may have subsided. A study found that at 1 year follow‐up, there was no significant difference in RV dimensions in those with and without COVID‐19 pulmonary embolism (Ilardi et al., 2023). Although, in the present study, RV s’ velocity was statistically significantly higher in the non‐COVID‐19 group than the COVID‐19 group, it was still within the normal range (Harkness et al., 2020). RV's velocity and TAPSE are a measure of longitudinal function whereas RVFAC is a measure of radial function (Hameed et al., 2023). Similar to the present study, Young et al. (2022) found RV‐GLS to be normal in 259 individuals post‐COVID‐19 and there was no clinically significant change in when serial echocardiograms were performed (Young et al., 2022).

**Figure 4:**
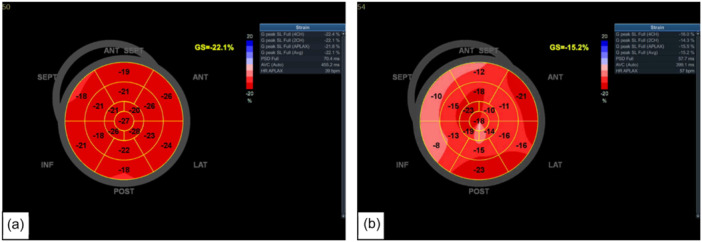
Left ventricular global longitudinal strain (LV‐GLS) measurements and bulls‐eye plots taken from two individuals of similar age without any underlying comorbidities (a) LV‐GLS of an individual with no history of coronavirus disease 2019 (COVID‐19) (non‐COVID‐19 group), (b) LV‐GLS of an individual with a history of COVID‐19 (COVID‐19) group.

**Figure 5:**
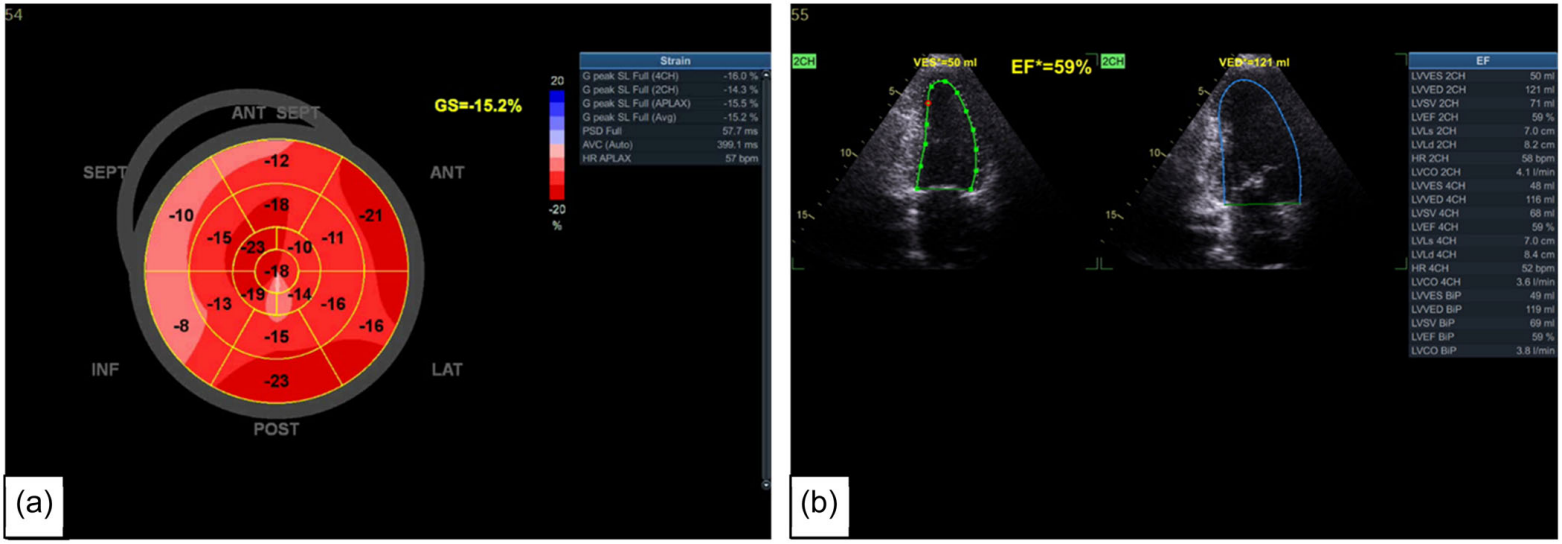
(a) Left ventricular global longitudinal strain (LV‐GLS) and bulls‐eye plot, (b) left ventricular ejection fraction (LVEF). Demonstrates an individual with a history of coronavirus disease 2019 (COVID‐19) with no underlying comorbidities has a normal LVEF but a reduced LV‐GLS. This may indicate subclinical damage to the left ventricle.

There is growing evidence that the left heart is also affected by the virus (Dweck et al., 2020). The present study demonstrated that LA diameter was significantly increased in the COVID‐19 group compared with the non‐COVID‐19 group. Previous research has shown that LA diameter may serve as a marker for stroke and risk of mortality (Benjamin et al., 1995). It may also be an indicator for heart failure, myocardial infarction and hypertension (Tsang et al., 2006). Left atrial diameter has also been shown to have an independent association with all‐cause mortality in males and females. LA enlargement or remodelling could be a manifestation of LV diastolic dysfunction given the age of the study cohort or through direct endothelial damage by COVID‐19. It is important to highlight that despite LA stiffness index being higher in the non‐COVID‐19 group, this was still within the normal reference range, where preserved LA reservoir function was observed (Sugimoto et al., 2018). Previous studies have also suggested that higher LA stiffness index with normal LA reservoir function could be explained by physiological adaptation and LA remodelling (D'Ascenzi et al., 2015). The present study demonstrates that LV‐GLS is significantly lower in the COVID‐19 compared with the non‐COVID‐19 group despite having normal LVEF (Figure 4). Most people have normal LV systolic function by traditional echocardiographic measures, despite having abnormal biomarker levels, cardiac magnetic resonance imaging findings and autopsy results (Freaney et al., 2020). LV‐GLS is not just a response to acute and critical illness and a short‐term response to viral infection, but an alteration which is persistent and sustained over time (Dalla et al., 2015). Previous research conducted on middle‐aged individuals with COVID‐19 suggested that reduced LV‐GLS may be a result of subclinical damage to the myocardium caused by COVID‐19 (Bhatia et al., 2021). Recent studies in a middle‐aged population found that those in the convalescent phase of COVID‐19 had lower LV‐GLS despite normal LVEF compared with age and gender‐matched healthy controls (Oikonomou et al., 2023; Turan et al., 2021). These results align closely with that of the present study; however, the study groups were relatively small compared with the present study. Another recent study in a large cohort of 595 participants found LV‐ and RV‐GLS to be reduced in 5.7% and 3.1%, respectively. However, the study included participants with comorbidities, underlying cardiovascular conditions and smokers (Garcia‐Zamora et al., 2023). This is a limitation that the present study has overcome by excluding many pre‐existing conditions that may alter cardiac function. Figure 5 shows that in the same individual with normal LVEF also had significantly lower LV‐GLS. Although LVEF is commonly used and is a very valuable tool in assessing cardiac function, it is more advantageous to use GLS, as it is more reproducible and directly measures cardiac contractility and detects subtle changes in cardiac contractility which can have therapeutic and prognostic benefits (Smiseth et al., 2016). Despite reduced LV‐GLS in the COVID‐19 group, an increase in SV and relative wall thickness was observed. This could be potentially explained by a compensatory mechanism to maintain CO, SV is increased through increasing myocardial wall thickness (Kamalov et al., 2010). However, it must be considered that despite LV‐GLS being reduced in the COVID‐19 group, there was no significant difference in LVEF between the two groups. End‐diastolic volume was also higher in the COVID‐19 group compared with the non‐COVID‐19 group. A mechanism to increase SV, thus maintaining CO, could be explained by increased filling time and increased venous return (Kamalov et al., 2010). Previous studies have found that changes in loading can cause a reduction in LV‐GLS, irrespective of myocardial status (Orru D'Ávila et al., 2023).

VAC has been associated with symptoms, functional performance and prognostic benefits in cardiovascular disease (Ikonomidis et al., 2019). There was no significant difference in VAC between the COVID‐19 and non‐COVID‐19 groups. VAC is affected by age and hypertension; two factors which were similar between both groups in the study and a likely explanation for our results (Ikonomidis et al., 2019). To date, there is a lack of evidence to suggest how the LV and arterial system interact in those affected by COVID‐19. A previous study showed that at 1‐month and 6‐month post‐COVID‐19, VAC was reduced in convalescent COVID‐19 patients compared to controls (Oikonomou et al., 2023). However, this study also included patients who were current smokers and with coronary artery disease. This makes it uncertain whether the reduced VAC is a result of COVID‐19 or whether this is due to smoking or the underlying coronary artery disease. (Central Illustration 1).

**Central Illustration 1:**
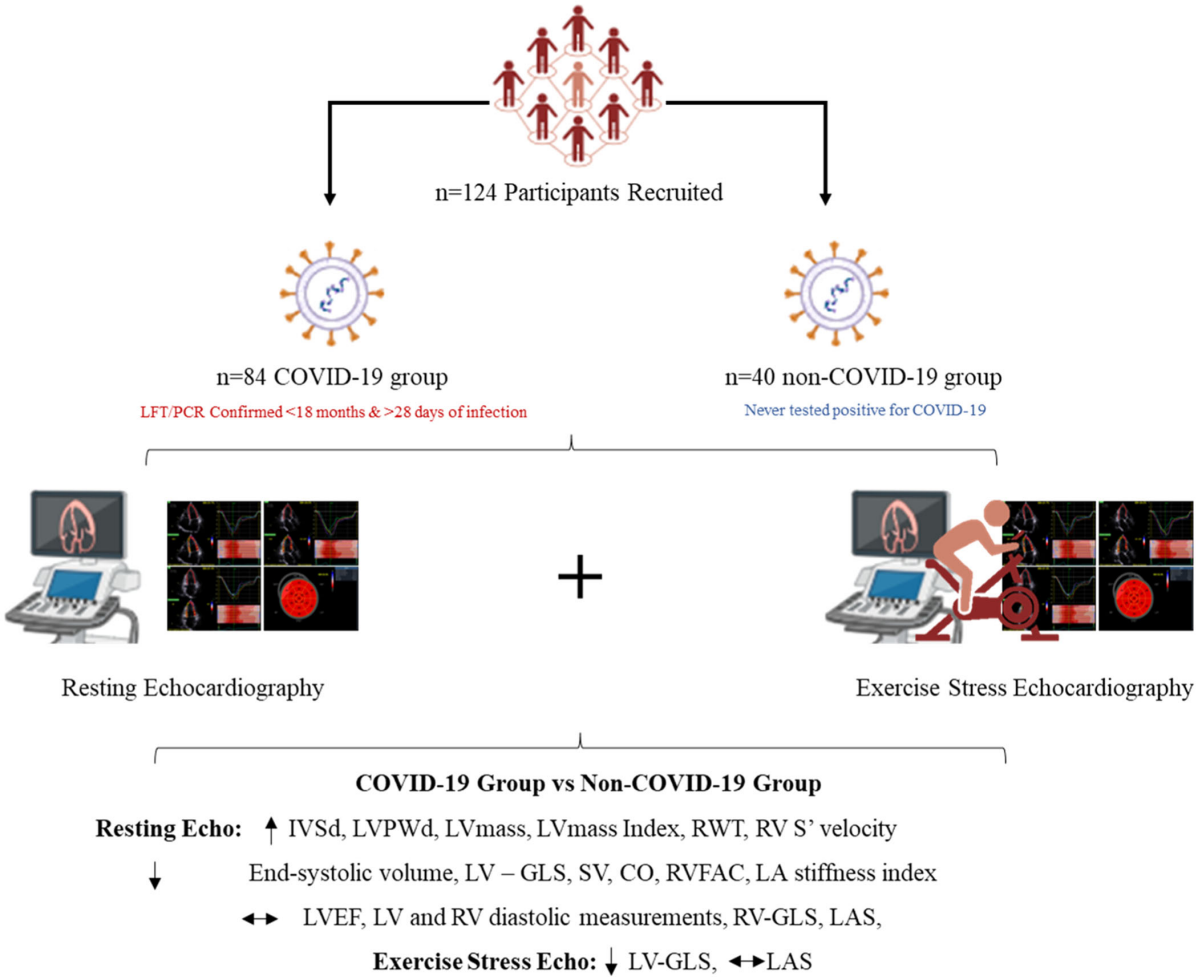
Coronavirus disease 2019 (COVID‐19) and the heart: The aim of the study was to evaluate the effects of COVID‐19 on cardiac structure and function in middle‐aged and older individuals. Those with a confirmed history of COVID‐19 demonstrated a higher left ventricular (LV) mass and relative wall thickness and left atrium (LA) diameter and significantly lower left ventricular global longitudinal strain (LV‐GLS), LV end‐systolic volume, cardiac output, right ventricle (RV) fractional area change and RV s’ velocity at rest compared with those who never tested positive for COVID‐19. Those with a history of COVID‐19 also demonstrated a lower LV‐GLS during peak‐exercise. COVID‐19 may be associated with cardiac structural and functional remodelling. Therefore, screening and monitoring may be beneficial post‐COVID‐19. CO, cardiac output; IVSD, intraventricular septum diameter in diastole; LAS, LA strain; LVPWd, left ventricular posterior wall diameter in diastole; LVEF, left ventricular ejection fraction; RVFAC, RV fractional area change; RWT, relative wall thickness; SV, stroke volume.

## 5 LIMITATIONS

The study has the following limitations. First, we cannot rule out the possibility that participants in the non‐COVID‐19 group did experience asymptomatic COVID‐19. The effects of the different variants of COVID‐19 on outcomes also could not be verified. Although the possibility of underlying co‐morbidities was reduced, we cannot confirm whether the results observed were primarily due to COVID‐19 or potential other clinical manifestations. This study was conducted in a middle‐aged and older population therefore the results cannot be generalized across all age groups. The effects of different variants on the cardiac structure and function remain unclear. Further research is warranted to elucidate possible mechanisms responsible for the changes observed.

## 6 CONCLUSION

The findings from this study suggest that middle‐aged and in older individuals with a history of COVID‐19 may demonstrate structural and functional remodelling of the heart. COVID‐19 disease may cause subclinical alterations in cardiac function even in those without pre‐existing diseases. Therefore, cardiovascular screening and monitoring of individuals post‐COVID‐19 may be beneficial to assess cardiac function, determine the risk of deterioration, and deploy interventions that may improve cardiac function.

## AUTHOR CONTRIBUTIONS

**Mushidur Rahman:** Formal analysis; investigation; writing—original draft; writing—editing. **Sophie L. Russell:** Investigation; writing—review and editing. **Nduka C. Okwose**: Formal analysis; investigation; writing—review and editing. **Helen Maddock:** Writing—review and editing. **Gordon McGregor:** Writing—review and editing. **Prithwish Banerjee:** Writing—review and editing. **Djordje G Jakovljevic:** Conceptualisation; funding acquisition; investigation; methodology; project administration; writing—review and editing.

## CONFLICT OF INTEREST STATEMENT

The authors declare no conflict of interest.

## Supporting information

Supporting information.

## Data Availability

Deidentified participant data generated from this research study will be shared upon reasonable request made to the corresponding author.
