# Supplementary material for: COVID‐19 is associated with cardiac structural and functional remodelling in healthy middle‐aged and older individuals
Source: Clin Physiol Funct Imaging. 2024 Oct 8;45(1):e12909. doi: 10.1111/cpf.12909 (PMC11650431; doi:10.1111/cpf.12909)
Supplement: Supplementary file 1 — Supporting information. [file CPF-45-0-s001.docx]

Left ventricular Mass was calculated using the cube formula published by the American Society of Echocardiography and European Association of Cardiovascular Imaging^1^.

LV mass = 0.8 [1.04(IVS + LVID + PW)^3^ – LVD^3^] + 0.6g

Left ventricular stiffness index was derived from the lateral mitral e’ velocity and the end-diastolic volume using the following calculation and a normal value of <0.111ml^-1^ was used^2^:

*LV Stiffness Index* =

End-diastolic volume

Transmitral E: Lateral mitral e’ velocity

Left atrial stiffness index was calculated as a radio from the following calculation and a normal value of 0.13-0.29 was used^3^:

*LA Stiffness Index* =

Left atrial reservoir strain

E/e’ average

Ventricular-arterial coupling was derived by firstly calculating arterial elastance (Ea) and ventricular elastance (Ees)^4^.

Ea = 0.9 x systolic blood pressure/stroke volume

Ees = 0.9 x systolic blood pressure/ end-systolic volume

VAC = Ea/Ees

Where systolic blood pressure was determined from the baseline blood pressure, stroke volume is Doppler-derived and end-systolic volume is calculated using 2D images. A normal VAC value of 2.3 ± 1.0 mmHg/mL was used^4^.

1. Lang RM, Badano LP, Mor-Avi V, Afilalo J, Armstrong A, Ernande L, et al. Recommendations for cardiac chamber quantification by echocardiography in adults: an update from the American Society of Echocardiography and the European Association of Cardiovascular Imaging. J Am Soc Echocardiogr Off Publ Am Soc Echocardiogr. 2015 Jan;28(1):1-39.e14.

2. Ngiam JN, Chew NWS, Tan BYQ, Sim HW, Kong WKF, Yeo TC, et al. Novel Echocardiography-Derived Left Ventricular Stiffness Index in Low-Flow Versus Normal-Flow Severe Aortic Stenosis with Preserved Left Ventricular Ejection Fraction. Sci Rep. 2020;10(1):1–8.

3. Sugimoto T, Robinet S, Dulgheru R, Bernard A, Ilardi F, Contu L, et al. Echocardiographic reference ranges for normal left atrial function parameters: Results from the EACVI NORRE study. Eur Heart J Cardiovasc Imaging. 2018;19(6):630–8.

4. Ikonomidis I, Aboyans V, Blacher J, Brodmann M, Brutsaert DL, Chirinos JA, et al. The role of ventricular–arterial coupling in cardiac disease and heart failure: assessment, clinical implications and therapeutic interventions. A consensus document of the European Society of Cardiology Working Group on Aorta & Peripheral Vascular Diseas. Eur J Heart Fail. 2019;21(4):402–24.
